# Low-Temperature Heat Capacity of CsPbI_3_, Cs_4_PbI_6_, and Cs_3_Bi_2_I_9_

**DOI:** 10.1021/acs.jpcc.3c05846

**Published:** 2023-11-13

**Authors:** Andries van Hattem, Jean-Christophe Griveau, Eric Colineau, Anton J. E. Lefering, Rudy J. M. Konings, Anna L. Smith

**Affiliations:** † Radiation Science & Technology Department, Faculty of Applied Sciences, Delft University of Technology, Mekelweg 15, Delft 2629JB, The Netherlands; ‡ European Commission, Joint Research Centre, 76125 Karlsruhe, Germany

## Abstract

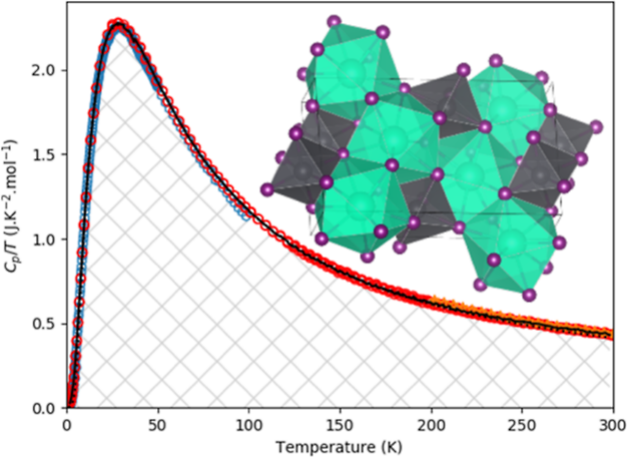

The heat capacities of CsPbI_3_, Cs_4_PbI_6_, and Cs_3_Bi_2_I_9_ were studied using low-temperature thermal relaxation calorimetry in the temperature range of 1.9–300 K. The three compounds are insulators, with no electronic contribution to the heat capacity. None of them show detectable anomalies in the studied temperature window. Thermodynamic properties at standard conditions are derived. Previously reported results on Cs_3_Bi_2_I_9_ are not fully consistent with the present findings. Moreover, the magnetic susceptibilities of the three title compounds were measured.

## Introduction

The compounds Cs_3_Bi_2_I_9_ and CsPbI_3_ were subjected to in-depth physical and chemical studies in fields varying from photovoltaics^1,2^ and scintillation^3,4^ to thermoelectrics^5,6^ because of the existence of a perovskite-related phase. Cs_4_PbI_6_ is the lesser investigated salt in the CsI–PbI_2_ system.^7−9^ In their review of metal halide perovskites, Kovalenko et al.^1^ describe the huge increase in publications in the field in the past decade. Despite the attention these materials are getting, studies of their low-temperature behavior seem to be rather limited and originate mainly from luminescence and scintillation-related studies.^7,10,11^ Other works include the investigation of thermal transport properties, reporting (ultra)low thermal conductivity for Cs_3_Bi_2_I_9_ and CsPbI_3_.^12−16^

The above-mentioned compounds, i.e., CsPbI_3_, Cs_4_PbI_6_, and Cs_3_Bi_2_I_9_, are the only stable phases that have been identified in the phase diagrams CsI–PbI_2_ and CsI–BiI_3_.^17−25^ Recently, the phase equilibria in the latter systems, together with the PbI_2_–BiI_3_ system, have been subjected to renewed research by several of the current authors, and a comprehensive CALPHAD model is now available, which describes the thermodynamic stability of the solid and liquid phases.^26^

The motivation for studying herein the low-temperature heat capacity of CsPbI_3_, Cs_4_PbI_6_, and Cs_3_Bi_2_I_9_ is given by the possibility of formation of these compounds in the case of a clad breach in a Lead-cooled Fast Reactor, a Generation IV type nuclear reactor.^27−30^ In case of a clad breach, the fission products cesium and iodine could come into contact with the coolant, which is either liquid Pb or a eutectic mixture of Pb and Bi (lead-bismuth eutectic). For the comprehensive thermodynamic assessment of the Pb–Bi–Cs–I system, thermodynamic and phase diagram information are necessary.

In this article, the low-temperature heat capacity up to room temperature is reported, providing insight into the physics and thermodynamics (heat capacity, entropy) of the complex metal iodides.

## Methods

### Synthesis and Pellet Preparation

Polycrystalline powders were synthesized as described by Van Hattem et al.^26^ Analysis of the powder X-ray diffraction data showed no secondary phases. The reader is referred to ref (26) for the corresponding Rietveld refinements and detailed analysis of the crystal structure. Samples were prepared by pressing the powders into pellets with a 3 mm diameter. The pellets used weighed 7.58(5) mg and 14.93(10) mg for CsPbI_3_, 5.17(5) mg and 10.05(10) mg for Cs_4_PbI_6_, and 3.14(5) mg and 11.70(10) mg for Cs_3_Bi_2_I_9_, respectively. For CsPbI_3_, the polymorph stable at room temperature and below, viz. δ-CsPbI_3_ was studied.

### Low-Temperature Heat Capacity Measurements

The low-temperature heat capacity was measured on two instruments, namely, a Physical Property Measurement System from Quantum Design (QD-PPMS), and a QD Versalab equipment system from QD. In the first case, the results were collected with zero field in the temperature range 1.9–350 K with slight variation of the precise temperature window per sample. In the second case, the data were collected with zero magnetic field in the temperature range 200–400 K. The samples were thermally connected to the puck platform using Apiezon N-grease or Apiezon H-grease, depending on the temperature range studied. First, a so-called addenda curve was measured, giving the heat capacity of the sample puck and the thermal grease. After the sample was loaded onto the puck, the total heat capacity was determined. The sample heat capacity was obtained by subtracting the addenda-curve from the total heat capacity, a procedure that is performed by the software of the instruments (Multiview from QD). All measurements were performed at a very high vacuum (10^–6^ mbar).

### Data Treatment

For a simple system, the lattice vibrations can be described with the Einstein model and the Debye model. For complex compounds, it is necessary to use a combination of the Debye and Einstein expressions if a model of a single Einstein or Debye expression fails to reproduce the lattice contribution to the low-temperature heat capacity data correctly by itself.^31,32^ Herein, a combination of one Debye model (*D*(Θ_D_, *T*)) and 1 Einstein model (*E*(Θ_E_, *T*) was found to be sufficient to fit the experimental data. The formula used for fitting in this work reads thus
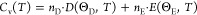
1where *n*_D_ + *n*_E_ should approximately be equal to the number of atoms in the formula and *C*_p_ ≈ *C*_v_ in the fitted temperature range. The parameters no longer represent the acoustic and optical branches but are rather to be understood as fitting parameters. The formulas for the Debye (with *x* = Θ_D_/*T*) and Einstein functions read, respectively
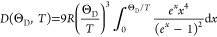
2
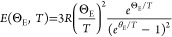
3

At low temperatures (typically below 10–20 K), the lattice contribution to the heat capacity is best fitted to a polynomial expression, which is merely a mathematical approach^33^
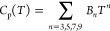
4

For CsPbI_3_, the Grüneisen parameter and thermal expansion coefficient are used as correction factors for the anharmonic effects beyond the Dulong-Petit limit, using ref (34) and substituting *C*_v_ = 3*nR*
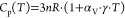
5where α_V_ is the thermal expansion coefficient and γ is the Grüneisen coefficient.

The Neumann–Kopp estimations^35^ made in this work use the following heat capacity values at 298.15 K, respectively: 52.47 J·K^–1^·mol^–1^ for CsI;^36^ 77.49 J·K^–1^·mol^–1^ for PbI_2_;^37^ and 82.2 J·K^–1^·mol^–1^ for BiI_3_.^38^

**Figure 1:**
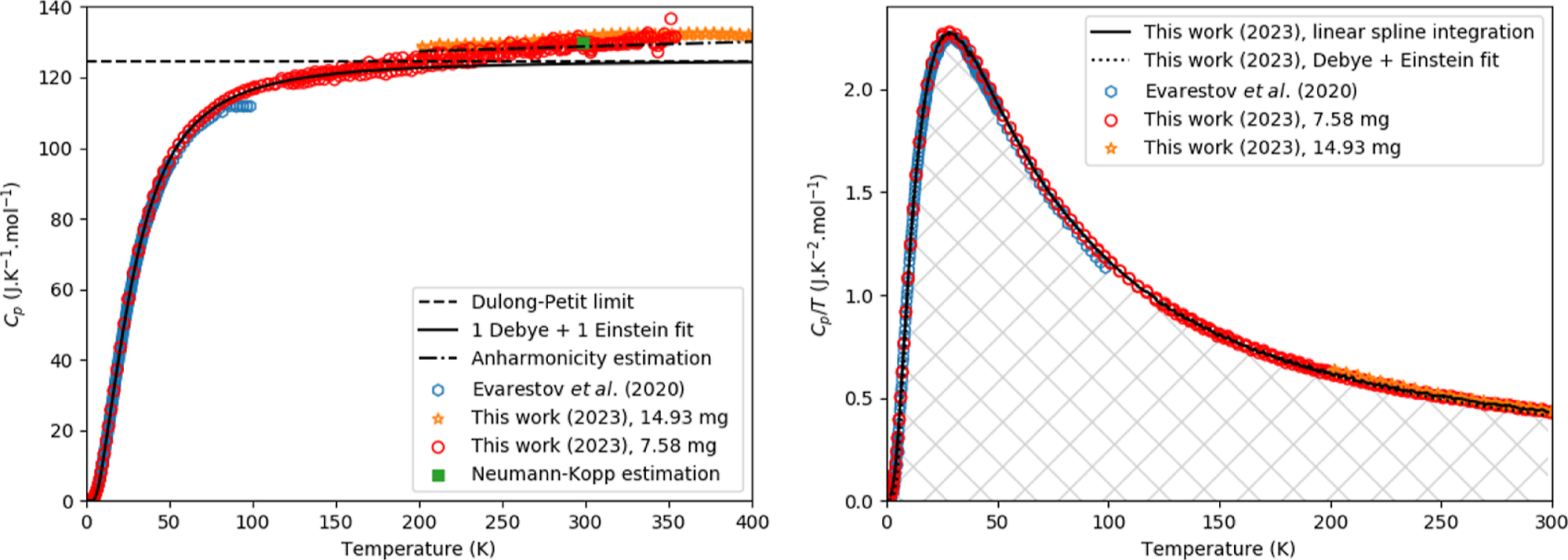
Experimentally measured heat capacity of CsPbI_3_ as measured in this work and compared with the Dulong-Petit law, the Neumann–Kopp estimation at 298.15 K, the data of Evarestov et al.,^40^ a calculation estimating the anharmonicity and a fit of 1 Debye and 1 Einstein function as plotted in *C*_p_ vs *T* (Left) and *C*_p_/*T* vs *T* (Right). The marked area in the right figure corresponds to the entropy as determined using linear spline (see main text).

The standard entropy value at 298.15 K reported herein was obtained using numerical integration of the data obtained with QD-PPMS using linear spline interpolation. The entropy contribution between 0 K and the lowest measured temperature point of each compound was calculated by the integration of a fit through the origin and the lowest measured points in the area between 0 K and the lowest measured temperature point. The entropy is indicated by the shaded area in Figures 1, 3 and 5, respectively.

**Figure 2:**
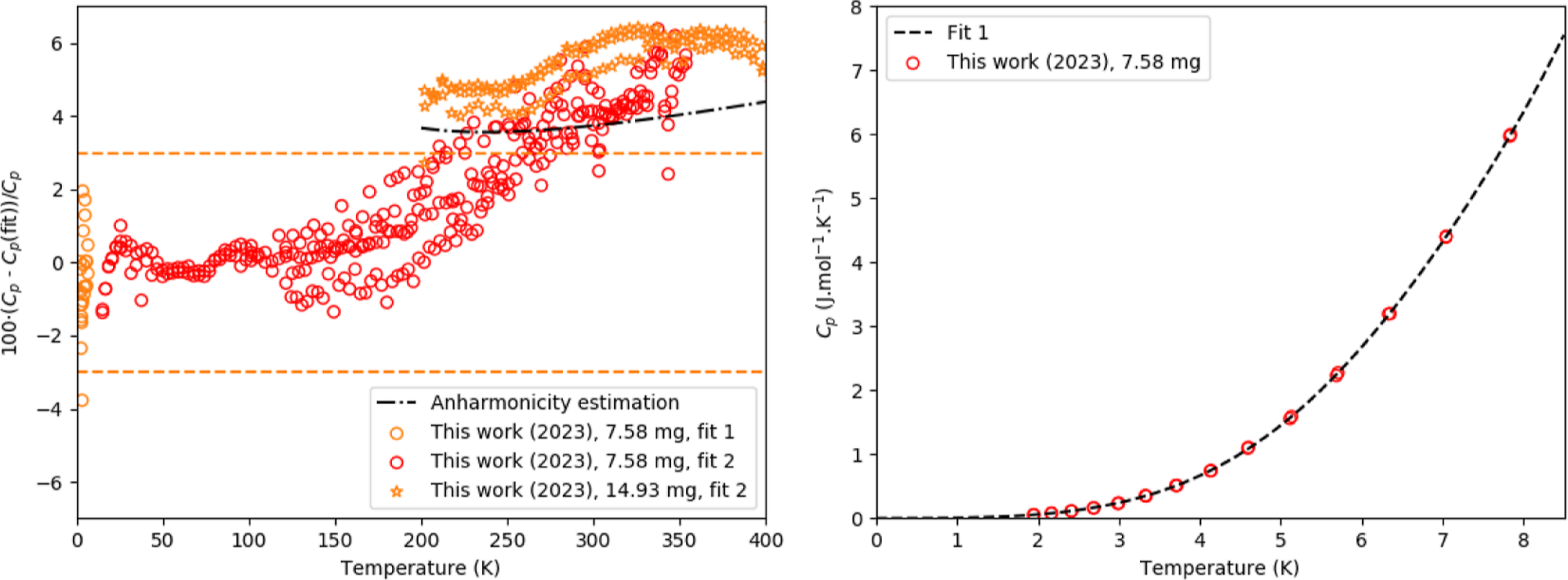
Left: Relative difference between experimentally measured heat capacity of CsPbI_3_ as measured in this work and fitting functions. Fit 1 is a fit by a polynomial equation at low temperature (eq 4); Fit 2 is the fit of one Debye and 1 Einstein function. The dashed orange lines are a guide to the eye and are drawn at a 3% deviation from the fit. Right: the low-temperature part fitted using eq 4 (fit 1).

**Figure 3:**
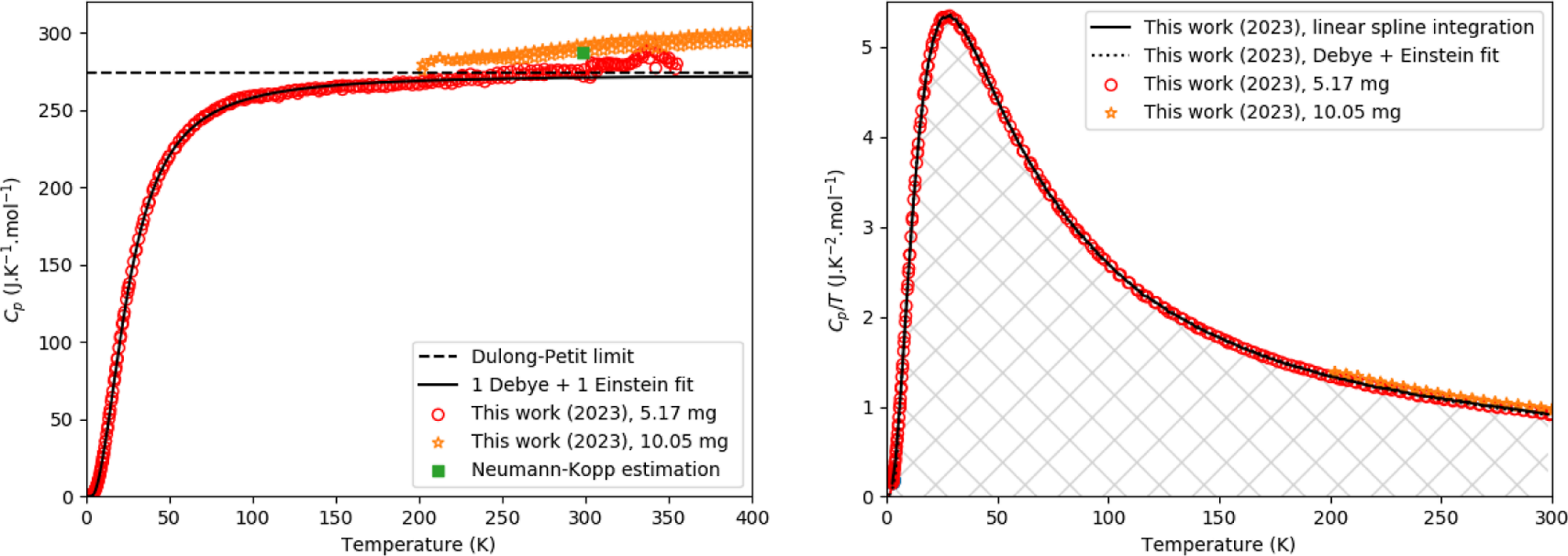
Experimentally measured heat capacity of Cs_4_PbI_6_ as measured in this work and compared with the Dulong-Petit law, the Neumann–Kopp estimation at 298.15 K, and a fit of 1 Debye and 1 Einstein function as plotted in *C*_p_ vs *T* (Left) and *C*_p_/*T* vs *T* (Right). The marked area in the right figure corresponds to the entropy as determined using linear spline (see main text).

**Figure 4:**
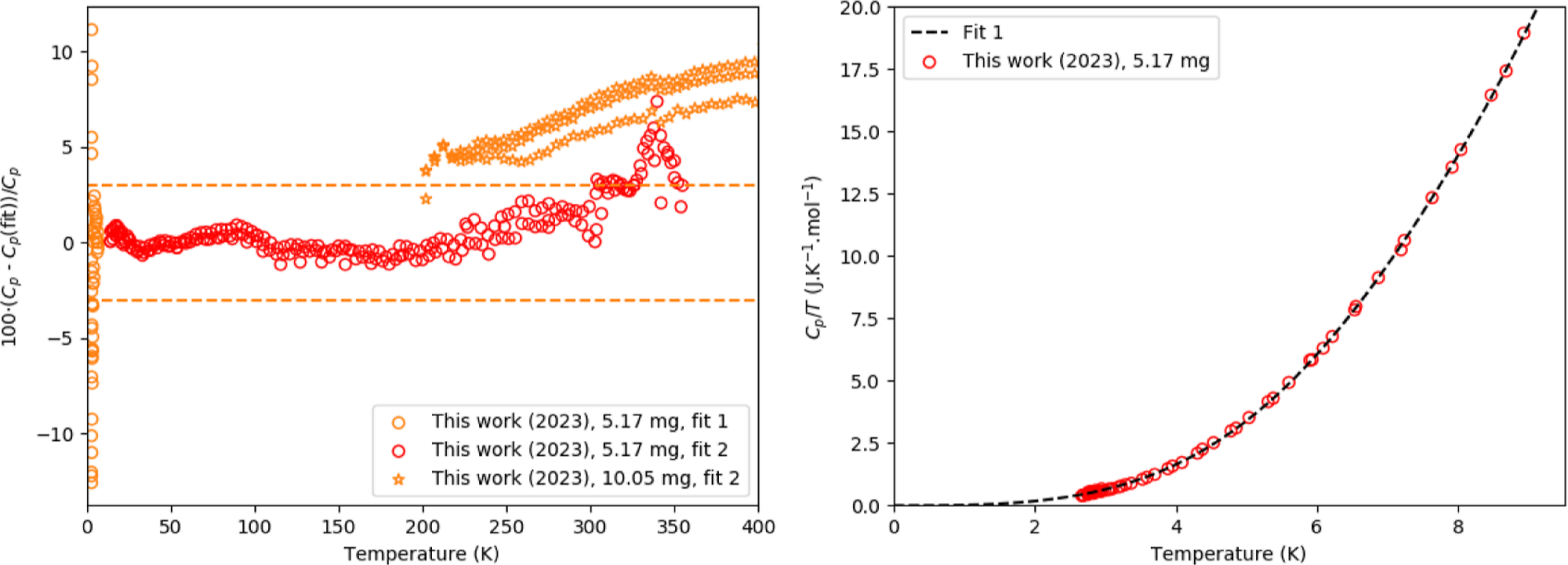
Left: Relative difference between experimentally measured heat capacity of Cs_4_PbI_6_ as measured in this work and fitting functions. Fit 1 is a fit by a polynomial equation at low temperature (eq 4); Fit 2 is the fit of one Debye and 1 Einstein function. The dashed orange lines are a guide to the eye and are drawn at a 3% deviation from the fit. Right: the low-temperature part, using eq 4 (fit 1).

**Figure 5:**
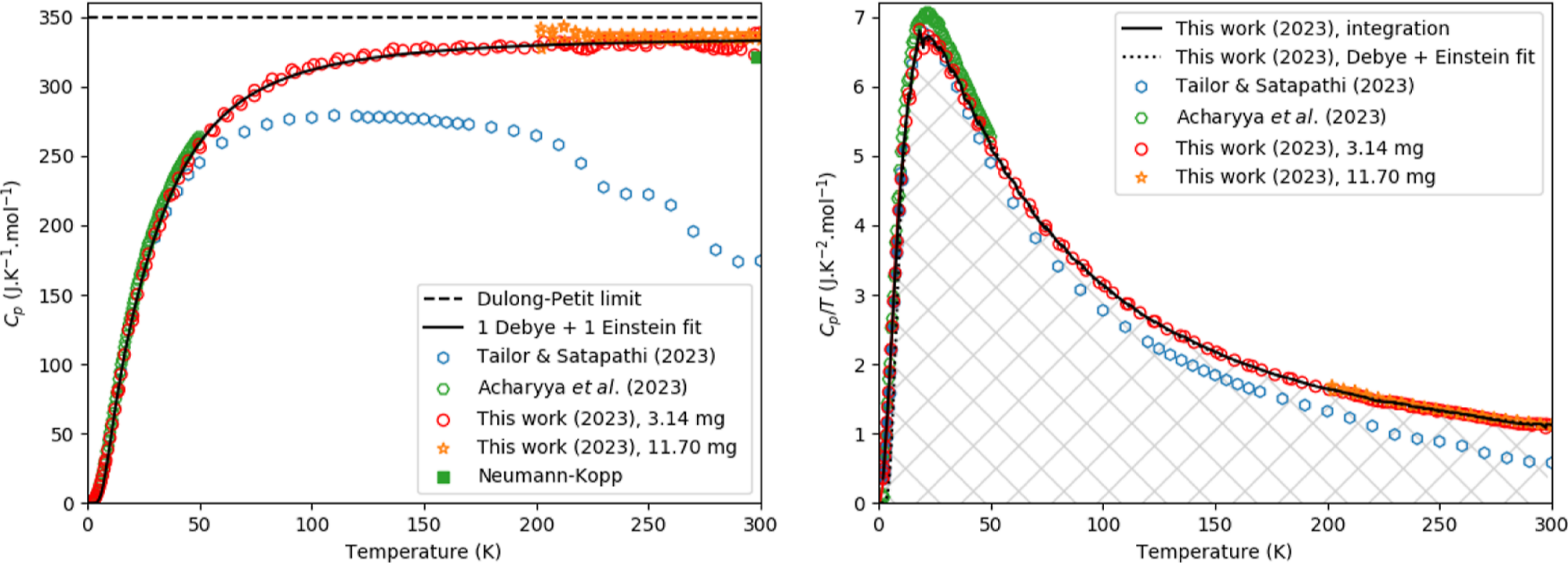
Experimentally measured heat capacity of Cs_3_Bi_2_I_9_ as measured in this work and compared with the data from Tailor and Satapathi,^54^ Acharryya et al.,^16^ the Dulong-Petit law, the Neumann–Kopp estimation at 298.15 K and a fit of 1 Debye and 1 Einstein function as plotted in *C*_p_ vs *T* (Left) and *C*_p_/*T* vs *T* (Right). The marked area in the right figure corresponds to the entropy as determined using a linear spline (see main text).

A 3% uncertainty on the measured heat capacity has been used in the error analysis.

### Magnetic Susceptibility Measurements

The static magnetic susceptibilities *M*/*H* of the three title compounds, CsPbI_3_, Cs_4_PbI_6_, and Cs_3_Bi_2_I_9_, respectively, were also measured on the MPMS-3 QD^39^ instrument by magnetic SQUID technique, achieving DC magnetization *M* from 2 to 300 K under constant magnetic field *H* up to 70 kOe. The samples used were identical to the heat capacity samples measured on the QD-PPMS instrument. The corresponding pellets had masses 6.61(5), 5.02(6), and 3.19(4) mg, respectively. Magnetization *M*(*T*) measurements were performed by thermalizing each piece of material using an exchange gas (6 N helium) at a partial pressure of 9 Torr. First, cooling was performed without a magnetic field applied (zero-field cooling). After the application of static DC magnetic field *H* = 70 kOe, each measurement point was then obtained by stabilizing the temperature *T* and subsequent extraction of the *M*(*T*) signal by mechanical oscillation.

## Results and Discussion

### Low-Temperature Heat Capacity of δ-CsPbI_3_

The experimentally measured heat capacity is shown in Figure 1, together with the Dulong-Petit limit, a Neumann–Kopp estimation from CsI and PbI_2_ at 298.15 K, the data of Evarestov et al.^40^ using QD-PPMS, a calculation estimating the anharmonicity and a fit obtained using one Debye and 1 Einstein function. The data obtained herein with the QD-PPMS and QD-Versalab instruments agree well.

The heat capacity rises fast; around 200 K, it already exceeds the Dulong-Petit limit. At 298.15 K, the experimentally measured value agrees with the value calculated using the Neumann–Kopp estimation rule (130 J·K^–1^·mol^–1^). In the lower temperature region, the heat capacity obtained in this work and the heat capacity obtained by Evarestov et al.^40^ agree perfectly, except for the last points measured between 90 and 100 K. In contrast to our results, the latter points seem to stabilize at a lower constant value.

For δ-CsPbI_3_, a correction using the Grüneisen parameter and thermal expansion coefficient (eq 5) is able to reproduce the heat capacity beyond Dulong-Petit fairly well. The Grüneisen parameter for this compound is reported to be 0.86^41^ and 1.03.^42^ Volumetric thermal expansion coefficients for δ-CsPbI_3_ of (132 × 10^–6^ K^–1^)^43^ and (118 × 10^–6^ K^–1^)^44^ have been determined based on cell parameters in the temperature region 298–609 K as determined using synchrotron-based powder diffraction. Using the average value and eq 5, a heat capacity value of 129.4 J·K^–1^·mol^–1^ is derived at 298.15 K, in agreement with the experimentally determined value and the Neumann–Kopp estimation. The calculation in the region 200–400 K using γ = 0.86 as shown in Figure 1 indicates that eq 5 models the heat capacity quite accurately. A calculation with γ = 1.03 is shown in Figure S.4. Analysis of the heat capacity toward 0 K shows that there is no electronic contribution to the heat capacity.

Fittings according to eqs 1 and 4 were made for δ-CsPbI_3_; the combined Debye–Einstein fit is shown in Figure 1, while the difference between both fits and the measured data is shown in Figure 2. The fitting parameters and temperature windows are listed in Table 1. In Figure 2, the dashed line indicates a 3% deviation;^45^ it can be seen that the error stays mostly within this margin; the extra deviation toward room temperature can be ascribed to anharmonic effects related to thermal expansion. In principle, the used technique can achieve a higher precision and accuracy,^46^ so a 3% uncertainty is used as a conservative error estimation.

**Table 1:** Fitting Parameters^a^

	CsPbI_3_	Cs_4_PbI_6_	Cs_3_Bi_2_I_9_
Harmonic Lattice Model			
temp. range (K)	2–8.5	2–9.5	2–14
γ (mJ·K^–2^·mol^–1^)	0	0	0
*B*_3_ (mJ·K^–2^·mol^–1^)	6.59 × 10^–3^	2.09 × 10^–2^	8.87 × 10^–2^
*B*_5_ (mJ·K^–4^·mol^–1^)	3.21 × 10^–4^	3.96 × 10^–4^	–6.63 × 10^–4^
*B*_7_ (mJ·K^–6^·mol^–1^)	–5.51 × 10^–6^	–6.64 × 10^–6^	2.89 × 10^–6^
*B*_9_ (mJ·K^–8^·mol^–1^)	3.00 × 10^–8^	3.19 × 10^–8^	–5.26 × 10^–9^
Debye and Einstein Fit			
temp. range (K)	8.5–135	14–275	14–298
*n*_D_ (mol)	3.583	9.39	6.75
Θ_D_ (K)	135.9	112.9	166.2
*n*_E_ (mol)	1.423	1.55	6.71
Θ_E_ (K)	44.6	38.0	44.8
*n*_D_ + *n*_E_ (mol)	5.006	10.94	13.46

a For explanation, see the main text.

The entropy and heat capacity at 298.15 K are calculated using linear spline interpolation in the measured region and extrapolation toward 0 K using the fitted eq 4. The obtained values are *S*_m_° (298.15 K) = (294.8 ± 8.9) J·K^–1^·mol^–1^ and *C*_p,m_ (298.15 K) = (128.6 ± 3.9) J·K^–1^·mol^–1^ as listed in Table 2. The obtained entropy value is around the lower limit given by Tsvetkov et al.;^47^ they estimate the entropy of CsPbI_3_ to lie in between 297.90 and 347.90 J·K^–1^·mol^–1^. The obtained heat capacity at 298.15 K is well in line with the Neumann–Kopp estimation.

**Table 2:** Thermodynamic Properties at 298.15 K

compound	*C*_p,m_ (298.15 K) (J·K^–^^1^·mol^–^^1^)	*S*_m_° (298.15 K) (J·K^–^^1^·mol^–^^1^) this work	*S*_m_° (298.15 K) (J·K^–^^1^·mol^–^^1^)^26^
δ-CsPbI_3_	128.6 ± 3.9	294.8 ± 8.9	294.8
Cs_4_PbI_6_	272.9 ± 8.2	659.5 ± 19.8	658.3
Cs_3_Bi_2_I_9_	338.2 ± 10.1	819.2 ± 24.6	819.2

For CsPbI_3_, several anharmonic features were reported in the recent literature. In 2020, Straus et al. reported single crystal X-ray diffraction and X-ray pair distribution function measurements on CsPbI_3_ in the temperature range 100–295 K.^48^ They found that the Cs atom occupies a single site from 100 to 150 K, above which thermal disorder results in two different Cs sites in the structure, one of which exhibits lower coordination by iodine anions. No phase transformation is associated with this. This implies that an anharmonic oscillator model would be more physically realistic above 150 K for CsPbI_3_. However, rattling effects occur usually at far lower temperatures, where they can be described with an Einstein-type model (single atom frequency vibrations) in a host lattice. Also, crystal-liquid duality or phonon glass-electron crystal phenomena, as discussed in relation to metal halide perovskites, seem to have no influence on the overall heat capacity.^49^ These theories describe the phonon behavior as a glass-like crystal, i.e., they are disordered and have no long-range correlation, and the atoms are not able to move well as compared to a liquid; this in contrast to a phonon liquid, which has a correlated movement of vibrational motion. Similar theories have been applied to CsPbBr_3_;^50,51^ the heat capacity of this compound also looks “classical”.^40^ Electron–phonon coupling is reported for CsPbI_3_ and explained by the 6*s*^2^-lone pair effect of Pb^2+^ by Huang et al.^52^ It is concluded that the reported anharmonic effects do not result in an anomaly in the heat capacity in the studied temperature range, though they do contribute to the increase of the heat capacity beyond the Dulong-Petit limit.

The magnetic susceptibility of CsPbI_3_ is perfectly diamagnetic and low in absolute value. It did not show any anomaly in the temperature range studied supporting heat capacity measurement results (Figure S.1). Interestingly, we obtained a much smaller value of magnetization than reported by Qiao et al.,^53^ suggesting the presence of magnetic impurities in their compound.

### Low-Temperature Heat Capacity of Cs_4_PbI_6_

The experimentally measured data are shown in Figure 3, together with the Dulong-Petit limit, the Neumann–Kopp estimation, and a fit of 1 Debye and 1 Einstein function. For Cs_4_PbI_6_, the Dulong Petit limit is reached at a higher temperature (around 270 K) than for CsPbI_3_. The data obtained with the QD-PPMS and QD-Versalab instruments are close, though the data obtained with the QD-Versalab are a bit higher. The Neumann–Kopp estimation is close to the measured values and in between both results. Toward the low-temperature part, a polynomial fit was used. The difference between the measured data and the combined polynomial and Debye–Einstein fit is shown in Figure 4. The fitting parameters are listed in Table 1. There is no electronic contribution to the heat capacity.

The entropy and heat capacity at 298.15 K are calculated using linear interpolation in the measured region and extrapolation toward 0 K. The obtained values are *S*_m_° (298.15 K) = (659.5 ± 19.8) J·K^–1^·mol^–1^ and *C*_p,m_ (298.15 K) = (272.9 ± 8.2) J·K^–1^·mol^–1^ as listed in Table 2. The error was again calculated using a 3% uncertainty on the heat capacity. To the best of our knowledge, no other investigations into the low-temperature heat capacity of Cs_4_PbI_6_ have ever been reported.

Here also, the heat capacity of Cs_4_PbI_6_ is in line with a diamagnetic, low in value, and quasi-temperature independent magnetic susceptibility, showing no anomaly as shown in Figure S.2.

### Low-Temperature Heat Capacity of Cs_3_Bi_2_I_9_

The experimentally measured heat capacity is shown in Figure 5, together with the Dulong-Petit limit, the Neumann–Kopp estimation at 298.15 K, the data of Tailor and Satapathi^54^ using QD-PPMS, Acharyya et al.^16^ (using QD-PPMS too) and a fit of 1 Debye and 1 Einstein function. Again, the heat capacity increases fast, though it does not exceed the Dulong-Petit limit in the studied temperature window. For the lower temperature part (till 50 K), the current data agree well with the literature data. At higher temperatures, the data in this work obtained with the QD-PPMS and QD-Versalab agree fairly well. Both measurements gave a heat capacity that slightly exceeds the value obtained using the Neumann–Kopp estimation rule.

**Figure 6:**
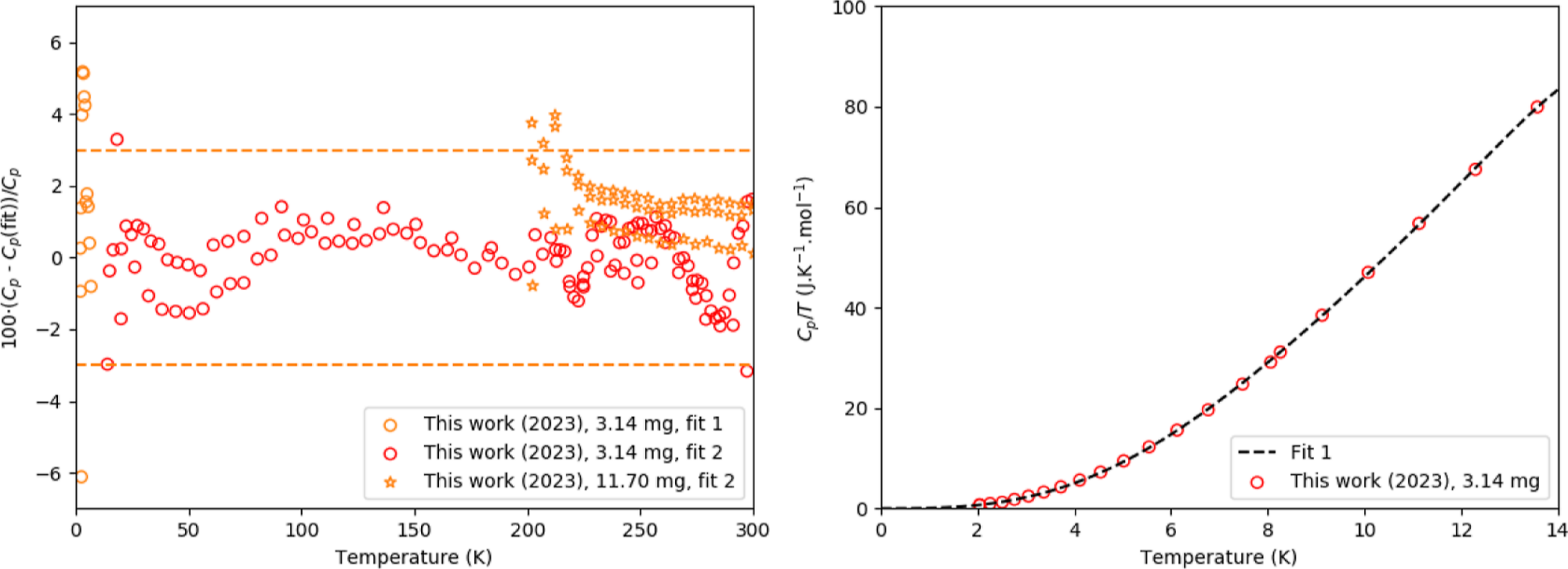
Left: relative difference between the experimentally measured heat capacity of Cs_3_Bi_2_I_9_ as measured in this work and fitting functions. Fit 1 is a fit by a polynomial equation at low temperature (eq 4); Fit 2 is the fit of 1 Debye and 1 Einstein function. The dashed orange lines are a guide to the eye and are drawn at a 3% deviation from the fit. Right: the low-temperature part, which shows a slight mismatch between the fitted and measured data.

The fits of eqs 1 and 4 are shown in Figures 5 and 6; the difference between the fits and measured data is shown in Figure 6. In this figure, the dashed line indicates a 3% deviation; it can be seen that the error stays mostly within this margin. The fitting parameters are given in Table 1.

**Figure 7:**
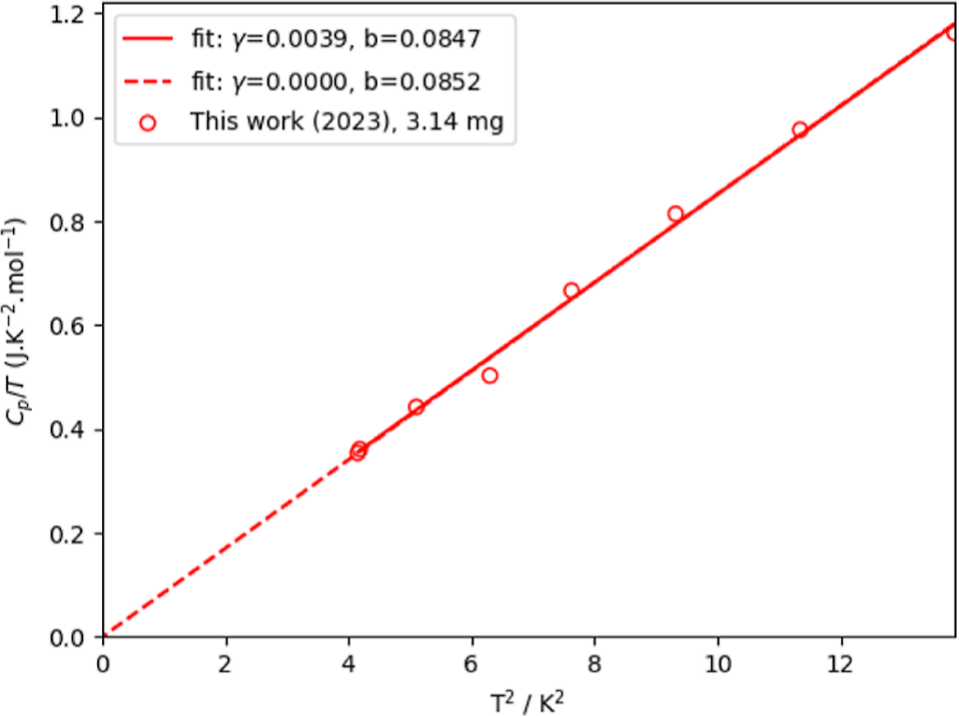
*C*_p_/*T* vs *T*^2^ in the lower temperature limit for Cs_3_Bi_2_I_9_ as fitted to *y* = γ + *b*·*x*, with an γ = 0 constraint for the second fit. The γ-term in the equation is the Sommerfeld coefficient.

There is no electronic contribution to the heat capacity, as can easily be concluded from Figure 7. This is in contrast with Tailor and Satapathi,^54^ who published a Sommerfeld coefficient γ = 0.762 J·K^–2^·mol^–1^. Clearly, this would be atypical for an insulating material. The Sommerfeld coefficient as published by Acharyya et al.^16^ (0.22 J·K^–2^·mol^–1^) is also unexpectedly high. In the first study, this is due to the low number of data points below 10 K, thus missing the typical curvature at low temperatures. In both studies, the mathematical fitting procedure involves a combination of Sommerfeld, Debye, and Einstein equations, in which case the derived coefficients lose their physical meaning. This resulted in very high reported coefficients and improper comparison with those of other materials.

Before the results of Tailor and Satapathi^54^ are further considered, a short overview of low-temperature studies is necessary for a thorough understanding of the low-temperature physics of Cs_3_Bi_2_I_9_. Its structure at 298 K was first solved in 1968.^55^ The low-temperature properties of this compound were studied for the first time by Melnikova et al. in 1996.^56^ They studied the optical birefringence and the dielectric and elastic constants and performed differential scanning calorimetry (DSC), besides nuclear quadrupole resonance (NQR). They observed a phase transition at 223 K based on the temperature dependence of the birefringence and the elastic constant, but an associated heat anomaly was too small to be detected with their DSC unit. They classify the phase transition as ferroelastic. Aleksandrov et al.^57^ continued the studies with ^127^I-NQR and reported an incommensurate phase in Cs_3_Bi_2_I_9_. They found that the second-order normal phase-incommensurate phase transition occurs at 220 K and reported the associated symmetry change. Another report by Melnikova and Zaitsev^58^ is a bit more elaborate and, besides reporting the same information, reports thermal expansion studies that lead to the conclusion that the phase transition is located at (220.0 ± 0.6) K. The next report is by Aleksandrova et al.^59^ They give a phase transition temperature of 224 K, based on ^127^I-NQR measurements. In 1999, Arakcheeva et al. reported the first attempt to solve the low-temperature phase using X-ray diffraction studies. This study was followed by a neutron diffraction study by Jorio et al.^60^ It needed another report by Arackcheeva et al. before the low-temperature structure was finally elucidated.^61^ It is stressed here that to solve the structure at room temperature down to the phase transition, anharmonic displacement parameters were necessary, while below the phase transition temperature, no anharmonic parameters were needed for a satisfying profile refinement. The anharmonic displacement parameters were especially useful in explaining residual electronic densities close to those of the Cs(1) and I(1) atoms. The authors hypothesize that the anharmonic displacements of these atoms, which are in highly symmetric positions, may be the driving force for symmetry breaking upon phase transition. Ivanov et al. further investigated Cs_3_Bi_2_I_9_ using ^133^Cs-NMR in 2001.^62^ In 2003, Girnyk et al.^63^ studied Cs_3_Bi_2_I_9_ in several ways: dilatometry, ultrasonic velocity, and domain structure, concluding to a first-order type phase transition. Based on thermal expansion studies, they calculated a latent heat associated with the phase transition around 220 K of 0.15 kJ·mol^–1^. Moreover, they found a thermal hysteresis of 5 K and identified the region from 183 until 221 K at cooling as heterophase, i.e., where the coexistence of the ferroelastic and paraelastic phases is claimed.

Between 2004 and 2010, a series of studies into the optical behavior of the low-temperature phase of Cs_3_Bi_2_I_9_ were furthermore reported by Motsnyi et al.^64−68^ Electron–phonon coupling was studied,^69^ as well as exciton–phonon coupling.^67,70^ Exciton–phonon interaction^67^ resulted in the disappearance of the layered structure below the transition temperature. Phonon calculations using VASP were performed by Geng et al.^12^ Until recently, no report on the low-temperature heat capacity of Cs_3_Bi_2_I_9_ had been published, apart from statements on an undetectable anomaly or very small latent heat by Melnikova et al.^56^ The recent work by Tailor and Satapathi^54^ is the first report of a heat capacity curve. The even more recent work by Acharyya et al.^16^ measured the heat capacity only up to 50 K. The discrepancy with respect to our own results for the electronic contribution was already discussed supra. Below, the contradictions between our results and the results of Tailor and Satapathi^54^ toward room temperature will be discussed.

Toward room temperature, the results are quite striking, as one can observe in Figure 5. The authors attributed this to crystalline-liquid duality in the material, but it is not clear to us how this could explain their results. Starting from low temperature, their results show a typical evolution for a heat capacity curve, in agreement with our data. However, a drastic deviation is observed starting above 50 K, ending via two steps at a value far below what is expected from classical limits. Their reference to disorder and anharmonicity would, in our view, result primarily in an increase in the heat capacity above the classical limit instead of a decrease. Given the literature on a phase transition around 220 K, a heat effect might be expected. However, no detectable heat effect was found, neither by Melnikova et al.^56^ nor in our work. Moreover, this could only explain a single anomaly in the heat capacity and not the shape of the heat capacity as reported by Tailor and Satapathi, which flattens already below 220 K and drops via two steps. Their attempt to explain their results refers to research into thermal conductivity. Cs_3_Bi_2_I_9_ is known to have a very low thermal conductivity (0.15 W·m^–1^·K^–1^ as measured on thin films)^12^ and cautiousness is necessary in measuring the heat capacity of insulating materials.^71^ When performing QD-PPMS measurements by the thermal-relaxation technique, caution should be taken with regard to the sample mass: for materials with a very low thermal conductivity, lower mass results in more accurate measurements; sample mass can affect the results already above quantities as small as 15 mg.^46^ No mass of the crystal is given in the work of Tailor and Satapathi,^54^ but based on the picture in their Supporting Information, the sample is estimated to be between 7 and 30 mg, so it is not clear to us whether the mass might play a role.

On top of the difference between the results on Cs_3_Bi_2_I_9_, it was also noted that the heat capacity for Cs_3_Bi_2_Br_9_, which also decreases unexpectedly in the report by Tailor and Satapathi, is not in line with previous measurements reported in the literature. Aleksandrova et al.^72^ measured the low-temperature heat capacity of Cs_3_Bi_2_Br_9_ and found a result in line with physical theory. To our surprise, Tailor and Satapathi even cite this paper but do not discuss the difference between their results and those of Aleksandrova et al. For all these reasons, we trust the results of Tailor and Satapathi on Cs_3_Bi_2_I_9_ only in the temperature window from 5 to 50 K and we have more confidence in the present results above that temperature.

As for the slight difference with the data as measured by Acharyya et al.^16^ their data have been extracted from a figure, thus introducing some error. In general, all data reported in the literature and measured in this work agree below 50 K, though the interpretation may differ (see discussion Sommerfeld coefficient above). Finally, the heat capacity of Cs_3_Bi_2_I_9_ does not show any anomaly, which is in line with its magnetic susceptibility from 2 to 300 K (Figure S.3).

Based on the current results, the entropy and heat capacity at 298.15 K are calculated using linear interpolation in the measured region and extrapolation toward 0 K. Using again an absolute error of 3% on the heat capacity, the obtained thermodynamic values are *S*_m_° (298.15 K) = (819.2 ± 24.6) J·K^–1^·mol^–1^ and *C*_p,m_ (298.15 K) = (338.2 ± 10.1) J·K^–1^·mol^–1^. These values are also listed in Table 2.

## Conclusions

The low-temperature heat capacities δ-CsPbI_3_, Cs_4_PbI_6_, and Cs_3_Bi_2_I_9_ were measured using a thermal-relaxation technique on two different instruments. These insulating materials showed no detectable anomalies in the studied temperature window. The standard entropy and heat capacity of the three compounds at 298.15 K have been derived, as listed in Table 2.

The obtained thermodynamic data were used to constrain the standard entropies of the ternary compounds in the model of the ternary salt system CsI–PbI_2_–BiI_3_ that was recently developed in our group in parallel to this work.^26^ The thermodynamic model optimization required only very slight to no deviation compared with the measured entropy data, as is shown in Table 2. Moreover, the magnetic susceptibility of the three compounds has been measured.

## Supporting Information Available

Supporting Information to “low-temperature heat capacity of CsPbI_3_, Cs_4_PbI_6_ and Cs_3_Bi_2_I_9_” (PDF)
Excel file containing the processed data of the heat capacity measurements (XLSX)
