# Supplementary material for: Low-Temperature Heat Capacity of CsPbI3, Cs4PbI6, and Cs3Bi2I9
Source: J Phys Chem C Nanomater Interfaces. 2023 Nov 13;127(46):22808–16. doi: 10.1021/acs.jpcc.3c05846 (PMC10684112; doi:10.1021/acs.jpcc.3c05846)
Supplement: Supplementary file 1 — jp3c05846_si_001.pdf [file jp3c05846_si_001.pdf]

Supporting Information to 'Low-temperature Heat Capacity of $CsPbI_3$, Cs_4PbI_6 and $Cs_3Bi_2I_9$'

Andries van Hattem,† Jean-Christophe Griveau,‡ Eric Colineau,‡ Anton J.E. Lefering,† Rudy J.M. Konings,† and Anna L. Smith*,†

†*Radiation Science & Technology Department, Faculty of Applied Sciences, Delft University of Technology, Mekelweg 15, Delft, 2629JB, The Netherlands*

‡*European Commission, Joint Research Centre, 76125 Karlsruhe, Germany*

E-mail: a.l.smith@tudelft.nl

Magnetic susceptibility $CsPbI_3$

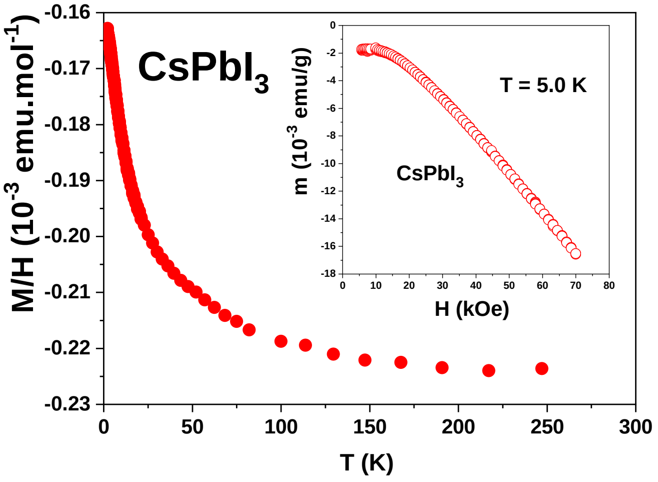

Figure S.1: ZFC Magnetic susceptibility of $CsPbI_3$ obtained at 70 kOe. Insert shows magnetisation dependence at fixed temperature 5.0 K.

Magnetic susceptibility Cs_4PbI_6

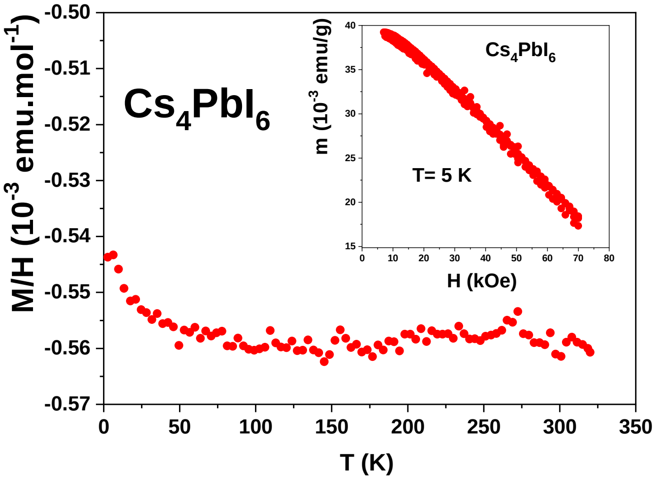

Figure S.2: ZFC Magnetic susceptibility of Cs_4PbI_6 obtained at 70 kOe. Insert shows magnetisation dependence at fixed temperature 5.0 K. A Ni impurity contribution ordered above 300 K is subtracted from results.

Magnetic susceptibility $Cs_3Bi_2I_9$

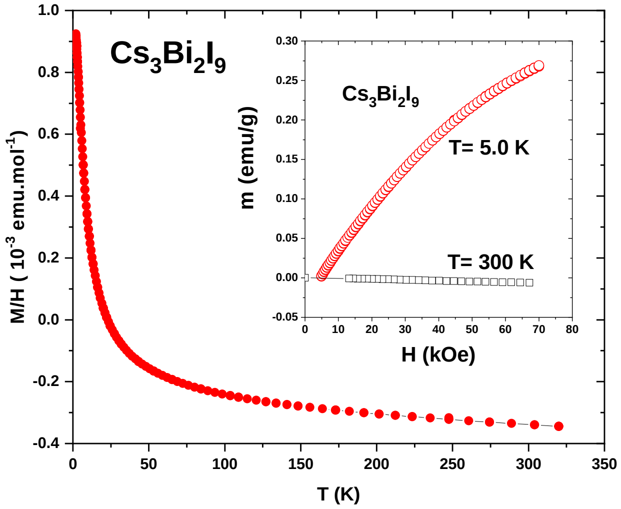

Figure S.3: ZFC Magnetic susceptibility of $Cs_3Bi_2I_9$ obtained at 70 kOe. Insert shows magnetisation dependence at fixed temperatures 5.0 K and 300 K. A Ni impurity contribution ordered above 300 K is subtracted from results.

Low temperature heat capacity of δ-$CsPbI_3$ with different Grüneisen parameter

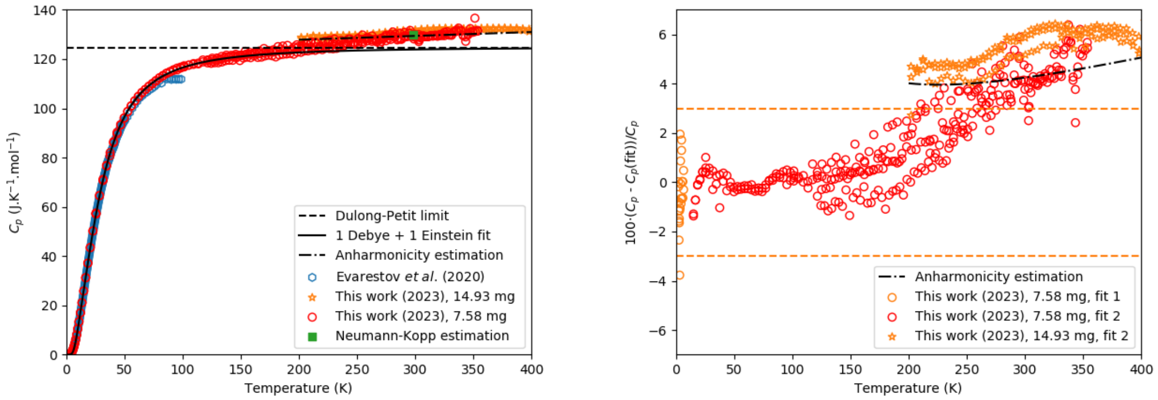

Figure S.4: Left: Low temperature heat capacity of $CsPbI_3$ with anharmonicity estimation based on a Grüneisen parameter of 1. Right: Difference of models and measured data.
